# Altered functional brain connectivity in patients with visually induced dizziness

**DOI:** 10.1016/j.nicl.2017.02.020

**Published:** 2017-02-28

**Authors:** Angelique Van Ombergen, Lizette Heine, Steven Jillings, R. Edward Roberts, Ben Jeurissen, Vincent Van Rompaey, Viviana Mucci, Stefanie Vanhecke, Jan Sijbers, Floris Vanhevel, Stefan Sunaert, Mohamed Ali Bahri, Paul M. Parizel, Paul H. Van de Heyning, Steven Laureys, Floris L. Wuyts

**Affiliations:** a Antwerp University Research Centre for Equilibrium and Aerospace (AUREA), University of Antwerp, Antwerp. Belgium; b Faculty of Medicine and Health Sciences, University of Antwerp, Wilrijk, Antwerp, Belgium; c Faculty of Sciences, Department of Physics, University of Antwerp, Antwerp, Belgium; d Coma Science Group, GIGA Consciousness, University and University Hospital of Liège, Liège, Belgium; e Neuro-otology Department, Division of Brain Sciences, Imperial College London, London, United Kingdom; f iMinds-Vision Lab, Department of Physics, University of Antwerp, Antwerp. Belgium; g Department of Otorhinolaryngology, Antwerp University Hospital, Edegem, Antwerp, Belgium; h Department of Radiology, Antwerp University Hospital, Edegem, Antwerp, Belgium; i KU Leuven – University of Leuven, Department of Imaging & Pathology, Translational MRI, Leuven, Belgium; j GIGA-Cyclotron Research Center – In Vivo Imaging, University of Liège, Liège, Belgium

**Keywords:** rsfMRI, Visually induced dizziness, VID, Functional connectivity, Vertigo, Vestibular

## Abstract

**Background:**

Vestibular patients occasionally report aggravation or triggering of their symptoms by visual stimuli, which is called visually induced dizziness (VID). These patients therefore experience dizziness, discomfort, disorientation and postural unsteadiness. The underlying pathophysiology of VID is still poorly understood.

**Objective:**

The aim of the current explorative study was to gain a first insight in the underlying neural aspects of VID.

**Methods:**

We included 10 VID patients and 10 healthy matched controls, all of which underwent a resting state fMRI scan session. Changes in functional connectivity were explored by means of the intrinsic connectivity contrast (ICC). Seed-based analysis was subsequently performed in visual and vestibular seeds.

**Results:**

We found a decreased functional connectivity in the right central operculum (superior temporal gyrus), as well as increased functional connectivity in the occipital pole in VID patients as compared to controls in a hypothesis-free analysis. A weaker functional connectivity between the thalamus and most of the right putamen was measured in VID patients in comparison to controls in a seed-based analysis. Furthermore, also by means of a seed-based analysis, a decreased functional connectivity between the visual associative area and the left parahippocampal gyrus was found in VID patients. Additionally, we found increased functional connectivity between thalamus and occipital and cerebellar areas in the VID patients, as well as between the associative visual cortex and both middle frontal gyrus and precuneus.

**Conclusions:**

We found alterations in the visual and vestibular cortical network in VID patients that could underlie the typical VID symptoms such as a worsening of their vestibular symptoms when being exposed to challenging visual stimuli. These preliminary findings provide the first insights into the underlying functional brain connectivity in VID patients. Future studies should extend these findings by employing larger sample sizes, by investigating specific task-based paradigms in these patients and by exploring the implications for treatment.

## Highlights

• Visually-induced patients present decreased functional connectivity of vestibular-related brain regions.
• Visually-induced dizziness patients present increased functional connectivity of visual and cerebellar brain regions.
• These findings might underlie typically seen symptoms in visually-induced dizziness, i.e. an overreliance on visual cues.
• This is the first exploratory study investigating the underlying neural aspects of visually-induced dizziness.
• These preliminary findings should be extended by larger sample sizes and by supplementing rsfMRI with task-based paradigms.

## 1 Introduction

A fundamental characteristic of mammals and humans is the ability to maintain gaze stabilization and postural control in normal circumstances (Goldberg et al., 2012). In order to do so, the human brain integrates visual, somatosensory and vestibular input (Goldberg et al., 2012). Depending on the circumstances and therefore the most relevant input, a central weighting favors one system more than the other (Peterka, 2002). In darkness for example, vestibular and somatosensory cues will dominate the less accurate visual information. This reweighting is done automatically and does not constitute problems, unless there is an underlying visual, vestibular or proprioceptive deficit (Peterka, 2002). In the latter, this might lead to dizziness, imbalance and falls. In the case of a peripheral vestibular lesion, some patients even develop an overreliance on visual cues, which might lead to visually induced dizziness.

Visual induced dizziness (VID) is characterized by the occurrence of vestibular symptoms as a result of complex or moving visual triggers, such as encountered during walking down supermarket aisles or the moving surroundings during driving (Page and Gresty, 1985, Jacob et al., 1989, Bronstein, 1995a, Bronstein, 1995b). VID is a term first implemented by the international classification committee of vestibular disorders (Bisdorff et al., 2009), but is also known as visual vertigo (Bronstein, 1995a, Bronstein, 1995b) or visual vestibular mismatch (Longridge et al., 2002). VID is a chronic disorder, often triggered by an acute vestibular disorder, during which these visual stimuli trigger or aggravate vestibular symptoms (Guerraz et al., 2001, Pavlou et al., 2006). Chronic vestibular symptoms triggered by an acute vestibular disorder can also manifest as chronic subjective dizziness (CSD) (Staab et al., 2004, Staab and Ruckenstein, 2005) or phobic postural vertigo (PPV) (Brandt, 1996, Kapfhammer et al., 1997), of which the former is also characterized by increased sensitivity to visual motion (Staab and Ruckenstein, 2005). When a group of 21 patients with VID were assessed for changes in postural sway and SVV in the presence of a tilted visual frame or a rotating visual disc, they displayed increased sway and poorer accuracy in estimating the gravitational vertical compared to controls - suggesting an influence of (moving) visual surroundings on vestibular processing (Guerraz et al., 2001). Later, a study by Pavlou and co-workers also reported that patients with VID show increased postural sway and worse results on the situational characteristics questionnaire (SCQ) when confronted with conflicting visual stimuli (Pavlou et al., 2006). Recently, our research group showed that visual roll motion is a crucial factor in provoking VID symptoms, which was also assessed by means of postural sway and questionnaires (Van Ombergen et al., 2016). The studies by Pavlou and Van Ombergen did not observe changes in SVV in challenging visual environments, whereas Guerraz and colleagues did. This discrepancy is most likely the result of a methodological difference and of inter-individual variability.

Most authors suggest that VID is the result of a defect in central reweighting of multisensory inputs (Bronstein, 1995a, Bronstein, 1995b, Guerraz et al., 2001), which is the phenomenon of adjusting the weight of different sensory modalities aiding in vestibular functions (e.g., postural control (Hwang et al., 2014)). In the case of VID patients, this means that the weight of visual input is too high, making these individuals strongly dependent on vision (Bronstein, 1995a, Bronstein, 1995b, Guerraz et al., 2001, Pavlou et al., 2006, Cousins et al., 2014, Van Ombergen et al., 2016). Indeed, this has been observed in patients with chronic vestibular symptoms after an acute vestibular neuritis, where symptom severity was associated with higher visual dependency (Cousins et al., 2014). However, it remains unclear whether these individuals acquired an increased visual dependency secondary to the vestibular insult or whether this was pre-existing, since it is a normally distributed trait in the general population (Witkin and Asch, 1948, Witkin, 1959). The former option would indicate a deficient sensory reweighting, where the visual system will account for the loss of vestibular function (Dieterich et al., 2007, Zu Eulenburg et al., 2010, Hong et al., 2014).

A recent structural MRI study of VID patients reported significantly more white matter abnormalities compared to dizzy controls without VID symptoms (Pollak et al., 2015). However, these changes were non-specific, therefore it is unclear where the white matter abnormalities were located and which white matter pathways they impinged upon. A separate study using fMRI with vestibular stimulation reported localized hypofunction and decreased connectivity between several brain regions including the superior temporal gyrus, anterior insula/inferior frontal gyrus, middle occipital gyrus and hippocampus in patients with chronic subjective dizziness (CSD) compared to controls (Indovina et al., 2015). The authors suggested that the VID symptoms, often present in CSD patients (Staab and Ruckenstein, 2005), might be related to the decreased connectivity between anterior insula and middle occipital gyrus together with the decreased activity in anterior insula, anterior cingulate cortex and hippocampus.

The current gaps in knowledge on the etiology and pathophysiology of VID highlight the need for further in-depth studies. We performed an explorative study, implementing resting-state fMRI analysis to study the brain's functional organization in rest. Resting-state fMRI has the advantage of reflecting the disease state more naturally, as opposed to task fMRI, where results are influenced by the choice of stimulus (e.g. Göttlich et al., 2014). We assessed differences in functional connectivity (i.e., the temporal correlation of the spontaneous BOLD response between spatially distant areas) between healthy control subjects and patients with VID using both hypothesis free and hypothesis-driven methods. For the latter, seeds belonging to the vestibular and visual networks were used.

## 2 Material and methods

### 2.1 Participants

Patients were recruited from the Department of Otorhinolaryngology at the Antwerp University Hospital. All patients underwent routine ear, nose, throat, and neuro-otological examinations, followed by specific audio-vestibular investigations when required. A detailed and systematic history was taken for each patient using the SO STONED questionnaire (Wuyts et al., 2016). Patients were included when showing a clear pattern of VID symptoms and triggers, based upon the questionnaire proposed by Mallinson for visual vestibular mismatch (Mallinson, 2011). Exclusion criteria were: 1) other medical conditions in the acute phase e.g. orthopedic injury, 2) fluctuating symptoms caused by episodic vestibular disorders (e.g. Meniere's disease) and 3) vestibular migraine. In addition, patients and control subjects were excluded if there were any contra-indications for the MRI examination.

In total, 10 VID patients were recruited (3 males, mean age (SD) 50.5 (8.3) years). As age- and gender-matched controls, 10 healthy participants (3 males, mean age (SD) 49.7 (6.1) years) were included. All participants were right-handed. Based on the history and/or results from the audio-vestibular test battery, a peripheral vestibular disorder was identified as the likely explanation for symptom onset in 9 out of 10 patients. In total, 5 patients presented with a unilateral vestibular hypofunction (two left, three right) and one patient presented with a bilateral areflexia. One patient presented with a unilateral vestibular hyperfunction left. One patient presented with abnormal low gain and phase for the vestibulo-ocular reflex. Two patients presented with an otolith dysfunction: one bilateral (concomitant with a unilateral horizontal semicircular canal hypofunction), one unilateral right. For three of the patients with a unilateral vestibular hypofunction, vestibular neuritis was identified as the specific etiologic diagnosis. For the other patients, a specific diagnosis could not be made since all of them were already in a chronic phase. Patients had persistent VID symptoms for 5.0 (3.1) years (mean duration (SD)), ranging from 1.2 to 9.9 years. None of the patients were assessed in an acute phase. For an overview, see Table 1.

**Table 1:** Summary of the demographic and clinical profile of the VID patients.

Patient	M/F	Age (years)	Disease duration (years)	Audio-vestibular tests	Etiology and disease status
Patient #1	F	39.7	4.4	Abnormal VOR	Unclear, chronic, uncompensated
Patient #2	M	56.6	3.5	Unclear	Unclear, chronic
Patient #3	F	48.3	6.0	R, otolith dysfunction	Unclear, chronic
Patient #4	M	57.9	3.9	L, hypofunction	VN, chronic, uncompensated
Patient #5	F	45.5	1.2	R, hypofunction	VN, chronic, uncompensated
Patient #6	F	52.2	9.9	BL, areflexia	Unclear, chronic
Patient #7	M	38.4	6.9	L, hyperfunction	Unclear, chronic
Patient #8	F	63.7	2.9	R, hypofunction	Unclear, chronic, uncompensated
Patient #9	F	56.9	1.4	R, hypofunction	VN, chronic, uncompensated
Patient #10	F	56.0	9.9	L, hypofunction BL, otolith dysfunction	Unclear, chronic

BL: bilateral; F: female; L: left; M: male; R: right; VN: vestibular neuritis; VOR: vestibulo-ocular reflex.

Ethical approval was provided by the local Ethics Committee of the University Hospital Antwerp (IRB number 13/38/357). Each participant provided a signed informed consent. All investigations have been conducted according to the principles expressed in the Declaration of Helsinki.

### 2.2 Data acquisition and analysis

Data was acquired on a 3 T scanner (Magnetom Trio Tim, Siemens AG, Siemens Medical Solutions, Erlangen, Germany) using a 32-channel head coil. The examination was performed with the patient in the following position: head first – supine. Earplugs were given to each subject and the head was stabilized with cushions to minimize head movement. The head was elevated 30° above horizontal to minimize the magnetic vestibular stimulation induced by the static magnetic field present within the scanner (Roberts et al., 2011). During the resting condition subjects were instructed to keep their eyes closed and refrain from structured thinking. During this period, 280 multislice T2*-weighted images were acquired with a gradient-echo echo-planar imaging sequence using axial slice orientation and covering the whole brain (voxel size = 3 × 3 × 3 mm^3^; matrix size = 64 × 64; slices: 42; repetition time = 2000 ms; echo time = 30 ms; flip angle = 77°; field of view = 192 × 192 mm). For anatomical reference, a high-resolution T1-weighted image was acquired for each subject (T1-weighted 3D magnetization-prepared rapid gradient echo sequence).

The three initial volumes were discarded to avoid T1 saturation effects. Data preprocessing was performed using Statistical Parametric Mapping 8 (SPM8; www.fil.ion.ucl.ac.uk/spm). Preprocessing steps included slice-time correction, motion correction, co-registration of functional onto its corresponding structural data, and Dartel-based spatial normalization to bring structural as well as EPI time series data in MNI space (MNI for Montreal Neurological Institute, http://www.bic.mni.mcgill.ca). EPI time series were slice time corrected and realigned. An additional intensity bias correction was applied in the fMRI data with the intensity bias map created during the segmentation of the mean fMRI image. The mean fMRI image was co-registered to its corresponding structural image before applying the co-registration parameters to the whole time series. fMRI images were then normalized to the MNI space using Dartel and smoothed with an isotropic 8-mm full-width at half maximum (FWHM) Gaussian kernel (Mikl et al., 2008).

Motion correction used an automatic artifact detection tool for global mean and motion outliers (http://www.nitrc.org/projects/artifact_detect/), these outliers were subsequently included as nuisance parameters (i.e. one regressor per outlier within the first-level general linear models) in the statistical analysis. Therefore, the temporal structure of the data was not affected. Specifically, an image was defined as an outlier (artifact) image if the head displacement in x, y, or z direction was > 0.5 mm from the previous frame, or if the rotational displacement was > 0.02 rad from the previous frame, or if the global mean intensity in the image was > 3 SDs from the mean image intensity for the entire resting session.

Analyses of functional connectivity were performed using the connectivity toolbox “CONN”, version 16B (http://www.nitrc.org/projects/conn; Whitfield-Gabrieli and Nieto-Castanon, 2012). Regression of nuisance effects before band pass filtering (RegBP; Hallquist et al., 2013) was used as recently recommended (Behzadi et al., 2007, Murphy et al., 2009, Saad et al., 2012, Wong et al., 2012). The data were despiked, and white matter (WM) and cerebrospinal fluid (CSF) components were regressed out as nuisance variables according to the aCompCor method (Behzadi et al., 2007). We then applied a linear detrending term. The residual BOLD time series went through a bandpass filter between 0.008 Hz and 0.09 Hz to reduce the effect of low frequency drifts and high-frequency noise. All described steps are part of the standard procedure in the “CONN” toolbox (Behzadi et al., 2007, Whitfield-Gabrieli and Nieto-Castanon, 2012). The residual head motion parameters were regressed out.

Two main analyses were performed. First we adopted a hypothesis-free (voxel-to-voxel) analysis. First-level voxel-to-voxel analysis encompassed the estimation of voxel-to-voxel functional correlation matrix within each subject. Bivariate correlation coefficients were computed from BOLD time series in every voxel within an a priori gray matter mask, from which the intrinsic connectivity contrast was computed. This characterizes the strength of the global connectivity pattern between each voxel and the rest of the brain. These results were assessed with an uncorrected height threshold of p = 0.001 together with an uncorrected cluster threshold of p = 0.01.

Second, a priori defined seeds were used in a seed-to-voxel approach.

We used seed regions within the visual system, specifically the associative visual cortex (10 mm spheres around x = − 30 y = − 89 z = 20 and x = 30 y = − 89 z = 20), secondary visual cortex (10 mm spheres around x = 10 y = − 6 z = − 78 and x = 10 y = 6 z = − 78), and primary visual cortex (6 mm spheres around x = 10 y = − 13 z = − 85 and x = 10 y = − 8 z = − 82).

Furthermore, we chose to investigate specific seed regions frequently associated with the distributed vestibular processing network, using coordinates taken from the extent literature. The posterior insula is an area of pivotal vestibular interest and long thought to be the human homolog to the monkey parieto-insular vestibular cortex (PIVC) (Dieterich and Brandt, 2001, Lopez et al., 2012, Zu Eulenburg et al., 2012). Thus, posterior insula and anterior insula segmentations were used (Kelly et al., 2012). The right parietal operculum 2 (rOP2) is a cytoarchitectonic area within the parietal operculum and was postulated as the primary candidate for the human vestibular cortex in a recent meta-analysis (Zu Eulenburg et al., 2012) (5 mm spheres around x = − 42 y = − 24 z = 18). The precuneus, a part of the multimodal human vestibular cortex was selected (Lopez and Blanke, 2011, Zu Eulenburg et al., 2012, Dieterich and Brandt, 2015) (10 mm sphere around x = 0 y = − 52 z = 27) and the inferior parietal lobule (IPL) which belongs to the multimodal human vestibular cortex (Lopez and Blanke, 2011, Lopez et al., 2012, Zu Eulenburg et al., 2012, Dieterich and Brandt, 2015) (10 mm spheres around x = − 51 y = − 51 z = 36 and x = 51 y = − 47 z = 42). Lastly, the bilateral vestibular nuclei (VN), receiving ipsilateral and contralateral afferent and efferent vestibular pathways, were chosen as ROIs (5 mm spheres around x = − 16 y = − 36 z = − 32 and x = 16 y = − 36 z = 32) (Miller et al., 2008, Kirsch et al., 2016). Additionally, seeds were placed in the thalamus, since they are a major relay station for vestibular signals (Lopez and Blanke, 2011) (4 mm sphere around MNI-coordinates x = 0 y = − 12 z = 9). Seeds with spheres around coordinates were made using the MarsBar toolbox (http://marsbar. sourceforge.net/).

For the bilateral seeds, time series of the left and right hemisphere were averaged together. These time series were then used to estimate whole-brain correlation r maps, which were then converted to normally, distributed Fisher's z transformed correlation maps to allow for subsequent group-level analysis. Two-sample *t*-tests were used to compare between the patients and control subjects. These results were reported as significant when they survived an uncorrected height threshold of p = 0.001 together with a family-wise error corrected extent threshold of p = 0.05 at the cluster level.

## 3 Results

The number of motion outliers did not differ between the two study groups (two-sided paired *t*-test; p = 0.98; mean (SD) patients = 18.6 (12.6); mean (SD) controls = 18.5(10.8).

**Fig. 1:**
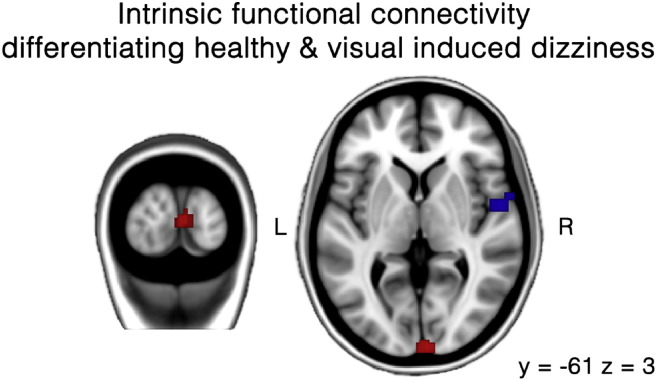
Differences in intrinsic functional connectivity between VID patients and healthy controls. Red regions indicate more intrinsic functional connectivity in VID patients, while the blue regions represent less intrinsic functional connectivity. Results were analyzed in a network-based manner and thresholded with an extended cluster level of p < 0.01. The MNI T1 template was used to render results. The (x, y, z) value indicates MNI coordinates of represented sections.

Intrinsic functional connectivity analysis compared VID patients and healthy controls. The results showed decreased functional connectivity in the right central operculum (superior temporal gyrus; STG) in VID patients, while an increased functional connectivity was found in the occipital pole (Table 2; Fig. 1).

**Table 2:** Intrinsic functional connectivity, hypothesis-free analysis.

	Peak voxels			Cluster size	Cluster p-unc	p-unc peak
	x	y	z			
**Controls vs. VID patients**						
Central opercular	54	− 4	− 4	229	< 0.0001	< 0.0001

**VID patients vs. controls**						
Occipital pole	6	− 100	6	87	0.006	< 0.0001

**Fig. 2:**
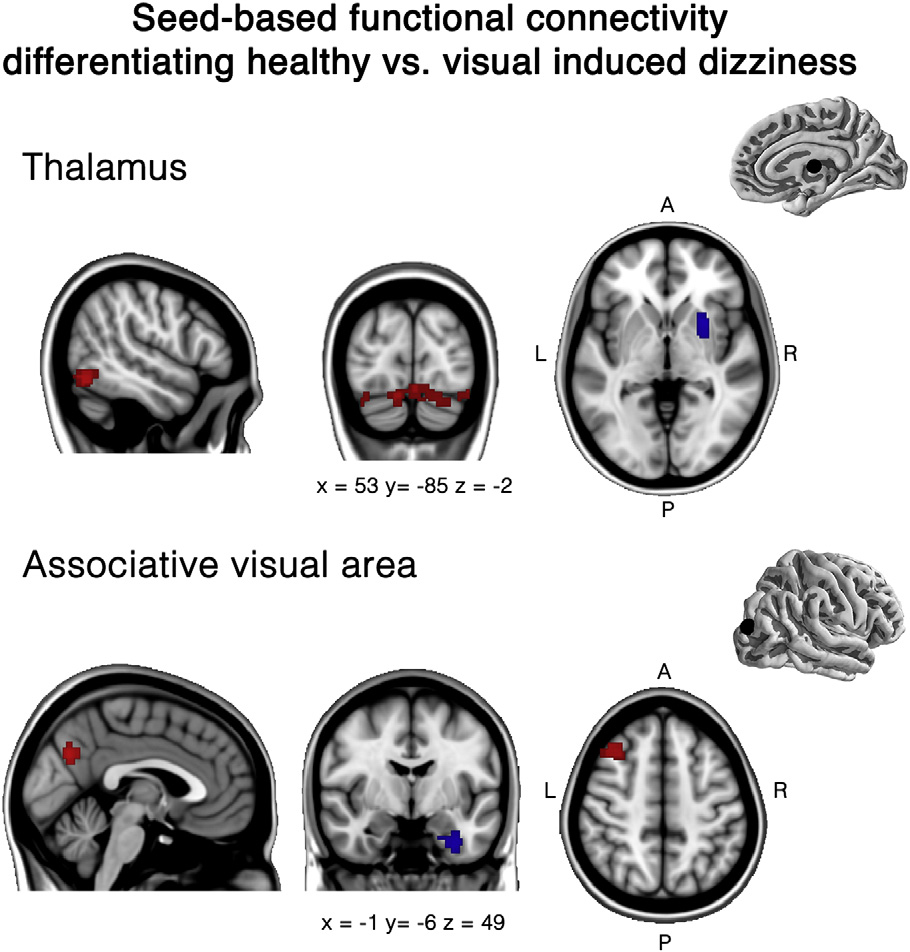
Differences in seed-based functional connectivity between healthy controls and VID patients. Two seeds showing significant differences between healthy controls and VID patients. Seed placement of the thalamus and associative visual areas are represented in the top right corner. Red regions indicate more intrinsic functional connectivity in VID patients, while the blue regions represent less intrinsic functional connectivity. Results were analyzed in a network-based manner and thresholded with a family-wise error corrected extended cluster level of p < 0.05. The MNI T1 template was used to render results. The (x, y, z) value indicates MNI coordinates of represented sections.

We found no significant differences between VID patients and controls for both the primary and secondary visual seeds with the seed-based analysis. However, we found significant differences in functional connectivity in the thalamus and associative visual cortex. VID patients show less functional connectivity between the thalamus and most of the right putamen (Table 3; Fig. 2). Furthermore, we found significantly less functional connectivity in VID patients between the visual associative area, overlapping with V5, and the left parahippocampal gyrus extending into the temporal pole (Table 3; Fig. 2).

**Table 3:** Controls vs. VID patients, seed-based analysis.

	Peak voxels			Cluster size	p-FWE cluster	p-unc peak
	x	y	z			
**Associative visual cortex**						
Left parahippocampal gyrus/temporal fusiform gyrus	− 30	− 4	− 36	255	0.032	< 0.0001

**Thalamus**						
Right putamen	28	12	0	290	0.015	< 0.0001

For the rOP2, IPL, insular, precuneus and VN seeds, we did not find significant differences between the VID patients and healthy controls. However, a significantly stronger functional connectivity between the thalamus and three clusters located in the cerebellum and occipital areas was found in VID patients. The largest difference was found in the central cerebellum 1 and 6 on the left, extending to the Crus1 and 2 on both left and right sides. Further differences were found in the right lateral occipital cortex extending to the inferior temporal gyrus (Table 4; Fig. 2). The third cluster of differences was found in its homolog on the left side, extending to the fusiform gyrus. Furthermore, we found significantly stronger functional connectivity between the associative visual areas and the middle frontal gyrus, as well as with the precuneus (Table 4; Fig. 2).

**Table 4:** VID patients vs. controls, seed-based analysis.

	Peak voxels			Cluster size	p-FWE cluster	p-unc peak
	x	y	z			
**Associative visual cortex**						
Right middle frontal gyrus	34	20	54	302	0.015	< 0.0001
Precuneus	− 2	− 70	32	240	0.039	< 0.0001

**Thalamus**						
Medial cerebellum, crus 1 & crus 2	16	− 94	− 24	638	0.0001	< 0.0001
Right lateral occipital cortex	40	− 78	− 18	380	0.004	< 0.0001
Left lateral occipital cortex	− 32	− 78	− 24	325	0.009	< 0.0001

## 4 Discussion

The aim of the current explorative study was to assess functional connectivity in patients with visually induced dizziness and to pinpoint potential biomarkers. Hereto, resting-state functional connectivity was assessed in VID patients and age- and gender matched controls. With a hypothesis-free exploration, we found a decreased functional connectivity in the right central opercular region (superior temporal gyrus) and an increased functional connectivity within the occipital pole in VID patients, when compared with controls. In a secondary seed-based analysis, VID patients showed increased connectivity between the thalamus and the cerebellum as well as the occipital cortex, and also between the associative visual cortex and the middle frontal gyrus and precuneus. Decreased connectivity was seen between the thalamus and putamen and between associative visual cortex and parahippocampal gyrus in VID patients.

The intrinsic connectivity contrast analysis allows the investigation of connectivity of brain regions, or clusters of voxels, which are affected by a change in connectivity with the rest of the brain without an a priori hypothesis (Martuzzi et al., 2011). The results of this study show a decreased connectivity in the right central opercular (in the STG), and an increase in the occipital pole for patients with VID. A previous meta-analysis has suggested the cytoarchitectonic area of right operculum parietal 2 (OP2) as the key vestibular cortical area (Zu Eulenburg et al., 2012). Furthermore, the STG is known to play a pivotal role in vestibular and multimodal processing, as shown by both caloric (Bottini et al., 1994, Suzuki et al., 2001, Deutschländer et al., 2002, Fasold et al., 2002, Dieterich et al., 2003, Indovina et al., 2005) and galvanic (Bense et al., 2001, Fink et al., 2003, Stephan et al., 2005) vestibular stimulation neuroimaging studies. Since VID patients have impaired vestibular function, the reduced connectivity in this area could reflect a deactivated state and thus a lower degree of reliance on the vestibular system for higher order spatial processing in rest. Moreover, a previous resting-state fMRI study has also shown a lower bilateral connectivity in similar regions (i.e. the posterior insula and the parietal operculum) in bilateral vestibular failure patients (Göttlich et al., 2014). Furthermore, clinical investigations suggest these patients have an overreliance on visual triggers (Bronstein, 1995a, Bronstein, 1995b, Guerraz et al., 2001, Pavlou et al., 2006, Van Ombergen et al., 2016), which might be reflected by the increased connectivity between the primary visual cortex (occipital pole) and the rest of the brain. In addition, the involvement of the right central opercular/STG is consistent with the general notion of spatial functions being lateralised to the right hemisphere in right-handed individuals (Dieterich et al., 2003).

The associative visual cortex was chosen as a seed region because a large part of it includes the motion-sensitive area V5 (Zeki et al., 1991), often denoted as MT/V5, which is expected to be involved in VID. An increased resting-state connectivity was observed with the middle frontal gyrus (MFG) and the precuneus in VID patients. The MFG is part of the vast vestibular cortical network, as indicated by the interaction of visual and vestibular stimulation (Della-Justina et al., 2014), which is supported by the findings of other fMRI studies using galvanic or caloric vestibular stimulation who also showed involvement of this area (Lobel et al., 1998, Bense et al., 2001, Dieterich et al., 2003, Stephan et al., 2005). Thus, the increased resting-state connectivity between the associative visual cortex and the MFG might indicate a shift towards a more visually driven input to this region and might partly explain the increased visual dependency. The precuneus on the other hand has a wide range of cognitive functions requiring input of many different modalities. It receives input from various vestibular and multisensory cortical areas, such as the intraparietal sulcus, the inferior parietal lobe and the parietal operculum (Leichnetz, 2001). Indeed, some of the previously mentioned fMRI studies also revealed changes in activity in the precuneus (e.g. Bense et al., 2001, Dieterich et al., 2003). Another fMRI study using optokinetic stimulation showed an increase in blood oxygen level dependent (BOLD) response in both the bilateral associative cortices and the precuneus (Kikuchi et al., 2009). Thus, our results may suggest that the functional output generated by the precuneus, such as mental imagery and motor programming (Cavanna and Trimble, 2006), is influenced more by the motion-sensitive area V5/MT in these patients.

The same associative visual cortex seed showed a decrease in functional connectivity with the parahippocampal gyrus. This region is known to be involved in visuospatial processing and memory (Aminoff et al., 2007) and serves as a direct input station to the hippocampus (Powell et al., 2004). Research on the macaque brain revealed parahippocampal activity during visual motion (Sato and Nakamura, 2003), suggesting a possible link between the two. However, it remains open to question as to whether there is an actual functional link between the two regions in humans.

The thalamus has a role in central vestibular processing as well, which is evident by neuroimaging studies (Bense et al., 2001, Suzuki et al., 2001, Indovina et al., 2005, Stephan et al., 2005) and lesion studies (Dieterich and Brandt, 1993, Dieterich et al., 2005, Lee et al., 2005). The thalamus receives input from the vestibular nuclei and the cerebellum (Hitier et al., 2014). In this study, when the thalamus was chosen as seed region, we found increased connectivity in VID patients with the occipital cortex, the left cerebellum and the medial cerebellum, while a decreased connectivity was seen with the putamen. The specifically affected regions of the cerebellum in this study were crus I and crus II. These are structures known to be involved in executive control (Habas et al., 2009), but also in navigation (Iglói et al., 2015) and visual attention (Kellermann et al., 2012). They are also functionally connected with the thalamus as is evident by a resting-state functional connectivity analysis (Sang et al., 2012), though what the exact role is of this connection in VID is still unclear.

The putamen is a part of the basal ganglia system, which has also been shown to activate after peripheral vestibular stimulation during fMRI acquisition (Bense et al., 2001, Dieterich et al., 2003, Stephan et al., 2005). A proposed role of the basal ganglia, and more specifically the putamen, in vestibular processing is that it uses vestibular input to create a motor outcome for posture control (Stiles and Smith, 2015). The connection between the vestibular system and the basal ganglia consists of a disynaptic pathway from the vestibular nuclei to the putamen through the thalamus (Lai et al., 2000). It is therefore possible that this connection is altered in VID patients. Another possibility is that the affected connectivity reflects a functionally impaired basal ganglia-thalamo-cortical loop, since Bense and coworkers attributed their finding of an activated putamen after vestibular stimulation to its involvement in the aforementioned feedback loop with the oculomotor cortex (Bense et al., 2001). This could possibly explain the hampered postural control in VID patients, as reported by several studies (Bronstein, 1995b, Guerraz et al., 2001, Pavlou et al., 2006, Cousins et al., 2014, Van Ombergen et al., 2016).

Our results are in line with the hypothesis that VID might result from a deficiency of central or sensory re-weighting, such that the visual weight remains higher than normal (Bronstein, 1995a, Bronstein, 1995b). Our results showed an increase in visual cortex connectivity and a decrease in vestibular cortex connectivity. We observed greater connectivity between visual motion area MT/V5 and the multisensory areas precuneus as well as the MFG, which might reflect this increased weight of visual information on higher order multisensory functions. This altered connectivity might underlie the poorer performance of VID patients when making judgements of subjective visual vertical (SVV) when a visual trigger is present, e.g. rod and disc or rod and frame tests (Guerraz et al., 2001). Patients will be influenced far more by what they see, such that they tend to align the SVV and adjust their posture more with the tilted frame, or with the direction of optical rotation, instead of relying on their vestibular information. The fact that the unbalanced sensory weight is observed for vestibular and visual stimuli is also consistent with the concept of reciprocal inhibitory visual-vestibular interaction (Brandt et al., 1998). This means that decreased vestibular function is automatically paired with decreased inhibition to the visual system, resulting in increased visual weight, which is seen in our results. Furthermore, it might be expected that this decreased vestibular function has an effect on vestibular cortical function. However, none of the vestibular cortical areas, which were chosen as seed regions, showed altered functional connectivity. A possible explanation is that these vestibular cortical areas do not exclusively receive vestibular input, but also somatosensory and visual input (Zu Eulenburg et al., 2012). Therefore, the contribution of these other sensory modalities might sustain intact functional connectivity of these brain regions. This has also been observed in an fMRI study, where patients with bilateral vestibular loss show enhanced visual activity, without vestibular regions being affected (Dieterich et al., 2007).

A factor that has to be considered when studying VID is the inter-individual heterogeneity of recovery from an acute vestibular disorder. For example, in the case of vestibular neuritis (VN), this can range from fully compensated to persisting symptoms after several years (Okinaka et al., 1993, Bergenius and Perols, 1999). A recent study found a positive correlation between visual dependency and subjective symptom assessment 6 months after the onset of VN (Cousins et al., 2014) and since visual dependency is also variable in the general population (Witkin and Asch, 1948, Witkin, 1959), the nature and expression of VID symptoms might well be different among patients. It has also been suggested that psychogenic factors, such as anxiety and introversion (Staab et al., 2014), or defects of the proprioceptive system (Bronstein, 1995a, Bronstein, 1995b) play part in the occurrence of VID as well. A limitation of the current study is the lack of psychophysical data such as assessments of visual dependency by means of subjective visual vertical (SVV) measurement. However, previous studies have already shown that SVV assessment does –in general – not correlate with other investigations in VID patients (Guerraz et al., 2001, Van Ombergen et al., 2016). Furthermore, our decision to use a healthy control group rather than a dizzy control group may have influenced the results. Future studies may wish to refine this approach by using larger sample sizes and by only including patients with matching aetiologies, and testing for differences between poorly compensated and non-symptomatic groups. However, this was beyond the scope of this investigation, therefore it is only possible to draw preliminary conclusions regarding the neural basis of VID. In addition, future studies should focus on specific task-based assessment of functional connectivity in VID patients. Examples hereof are vestibular stimulation (as implemented by (Indovina et al., 2015)), but also visual stimulation since these patients experience symptoms most frequently in specific visual environments. Furthermore, tasks which employ a combination of vestibular and visual stimulation (congruent with each other or incongruent, i.e. conflicting) could be an interesting paradigm to assess VID patients, e.g. Roberts et al. (2016).

However, to the best of our knowledge, this is the first report on the underlying functional brain connectivity in VID patients. Although preliminary, this study can help to focus and refine future studies. The brain areas where we observed differences are largely supported by the current literature on visual and vestibular processing. Nevertheless, some results are more difficult to interpret. For example, the crus I and crus II of the cerebellum show diminished connectivity with the thalamus and although there is evidence on their involvement in visuospatial tasks (Kellermann et al., 2012, Iglói et al., 2015), the functional link between the two regions relevant for visual-vestibular processing is not very clear. The same issue can be considered for the functional connectivity between the associative visual cortex and the parahippocampal gyrus. It should be noted that brain areas such as the precuneus and thalamus are thought to be involved in many different functions, making it difficult to assess precisely which functional part of the brain area is involved and it remains speculative what the observed altered connectivity reflects on the functional level. In addition, apart from extending the current results, future studies should focus on the implication of these findings for therapeutic options in VID patients. It has already been shown that rehabilitation programmes by means of optokinetic stimulation are beneficial for these patients (Pavlou et al., 2011, Pavlou et al., 2013). In addition, it should be investigated whether VID patients could potentially benefit from e.g. neuromodulation, which has been used to modulate and assess vestibular cortical processing (Ahmad et al., 2014, Arshad et al., 2014) and in the treatment of other vestibular pathologies (Cha et al., 2016).

## 5 Conclusion

In conclusion, we found alterations in the visual and vestibular cortical network in VID patients that could possibly explain why these patients show amplification of their vestibular symptoms when being exposed to complex and challenging visual stimuli. In addition, these findings could underlie this overreliance on visual cues, also defined as high visual dependency. Although preliminary, these findings provide first insights into the underlying functional brain connectivity in VID patients and might help to define biomarkers. Future studies could extend upon these preliminary findings by employing larger sample sizes and by supplementing resting-state fMRI investigations with specific task-based paradigms. In addition, the exact implication of these findings for possible therapeutic options should be assessed.

## Conflicts of interest

The authors declare no competing financial interests.
